# Assessment of Patient Satisfaction and Outcomes After Outpatient Joint Arthroplasty in Academic Medical Centers

**DOI:** 10.1016/j.artd.2023.101246

**Published:** 2023-11-10

**Authors:** Soham Ghoshal, Carlos Salazar, Jessica Duggan, Cole Howell, Antonia F. Chen, Vivek M. Shah

**Affiliations:** a Harvard Medical School, Boston, MA, USA; b Department of Orthopaedic Surgery, Brigham and Women’s Hospital, Boston, MA, USA; c Albany Medical College, Albany, NY, USA

**Keywords:** Total joint arthroplasty, Unicompartmental knee arthroplasty, Patient satisfaction, Outpatient, Academic medical center

## Abstract

**Background:**

There is limited literature evaluating patient satisfaction and outcomes after outpatient joint arthroplasty procedures in academic medical centers (AMCs). The aims of this study are to determine: (1) patients' desires to repeat their procedures and be discharged on the same day, (2) patient-reported outcome measures (PROMs), (3) time to discharge, (4) readmission rates, and (5) factors that predict PROMs in patients undergoing outpatient joint arthroplasty in AMCs.

**Methods:**

A prospective survey was completed by 66 total hip arthroplasty (THA), 35 total knee arthroplasty (TKA), and 180 unicondylar knee arthroplasty (UKA) outpatients who underwent surgery from May 2018 to December 2020 in 2 AMCs. The survey consisted of questions regarding hip or knee PROMs (Hip Disability and Osteoarthritis Outcome Score for Joint Replacement, and Knee Injury and Osteoarthritis Outcome Score for Joint Replacement), satisfaction with outpatient procedures and discharges, and reasons for readmissions. Linear regression analysis was conducted with statistical significance set at *P* < .05.

**Results:**

100% of THA, 93.8% of TKA, and 93.0% of UKA outpatients stated that they would re-elect to undergo their respective procedure. Furthermore, 94% of THA, 81% of TKA, and 95% of UKA patients stated they would like same-day discharge again. THA, TKA, and UKA patients reported respective mean PROM scores of 94.7, 89.9, and 86.1. Readmission rates were 1.5%, 0.0%, and 0.5%, for THA, TKA, and UKA, respectively.

**Conclusions:**

Patients who underwent outpatient joint arthroplasty procedures at 2 AMCs experienced minimal readmissions and reported a high desire to repeat their outpatient procedures.

## Introduction

Recent literature predicts that more than half of total joint arthroplasty (TJA) procedures in the United States will be performed on an outpatient basis by 2026, defined as discharge on the same day of surgery [1,2]. This shift may be attributed to concerns with staffing, time constraints, and financial pressures, as TJA procedures are increasingly done in ambulatory surgery centers (ASCs) [3]. Previous studies have reported satisfactory outcomes with TJA when performed under an ASC model [4]. A study performed by Berend et al. [5] reported that 98% of patients undergoing outpatient TJA procedures at their own ASC reported great or good satisfaction scores from 2014 to 2015. As ASCs are privately owned, there is often more control over staff and the entire TJA process to maximize patient satisfaction. In academic medical centers (AMCs), however, hospital employees are driven by different incentives and organizational structures, which may influence patient satisfaction differently.

An additional critical factor influencing patient satisfaction and outcomes at AMCs is the length of stay, as an increased length of stay has been shown to increase the risk for medical errors and complications [6]. As earlier discharges from hospitals tend to improve patient recovery metrics for those undergoing minimally invasive arthroplasty procedures, AMCs are increasingly incentivized to move toward same-day discharges for TJA procedures [[7], [8], [9], [10]]. Recent studies have demonstrated that outpatient TJA procedures performed at AMCs lead to fewer 90-day readmissions as well as decreased 30-day and 90-day postoperative costs when compared to inpatient TJA procedures [11]. Thus, a better understanding of patient satisfaction and outcomes following same-day total hip arthroplasty (THA), total knee arthroplasty (TKA), and unicondylar knee arthroplasty (UKA) procedures may guide AMCs to shift these procedures to an outpatient basis. However, to our knowledge, there have been no studies evaluating patient satisfaction and outcomes in patients undergoing outpatient TJA or UKA procedures at AMCs. Therefore, the present study aims to address this gap in the literature by (1) evaluating patient satisfaction defined as patients’ desires to repeat their procedures and be discharged on the same day, (2) patient-reported outcomes, (3) time to discharge, (4) readmission or reoperation rates in patients undergoing outpatient TJA or UKA in AMCs, and (5) whether a variety of patient or surgical factors can predict postoperative patient-reported outcome measures (PROMs). Given the high demonstrated satisfaction scores at ASCs, we hypothesize that patients undergoing same-day TJA procedures at AMCs will also report high patient satisfaction and positive PROMs.

## Material and methods

We conducted a prospective cohort survey study in outpatients who previously underwent TJA or UKA at 2 large academic quaternary care institutions between May 1, 2018, and December 31, 2020. Following institutional review board approval, adult patients (>18 years of age) were sent recruitment letters for the study through their online patient gateway portals. Informed consent was obtained, and patients were surveyed on their desire to repeat their surgery (patient satisfaction), experience with same-day discharge, and reasons for readmission or reoperation. Readmissions were identified in patient charts and confirmed through the survey. The survey is included in the Appendix.

Previous researchers have developed and validated tools for assessing PROMs following TJA procedures such as the Hip Disability and Osteoarthritis Outcome Score of Joint Replacement (HOOS, JR) and Knee Injury and Osteoarthritis Outcome Score for Joint Replacement (KOOS, JR) surveys [12]. In this study, THA PROMs were measured using the HOOS, JR survey and TKA and UKA PROMs were measured using the KOOS, JR survey at a minimum of 1 year postoperatively. The HOOS, JR and KOOS, JR surveys evaluate various PROMs including pain, stiffness, and difficulty when performing movements attributed to the hip or knee over the past week. These results were self-reported on a scale of 0-4 (none, mild, moderate, severe, extreme, respectively). The HOOS, JR and KOOS, JR surveys were subsequently scored by summing the raw responses (ranges 0-24 and 0-28, respectively) and then converting it to an interval score (range 0-100), where 0 represents total hip or knee disability and 100 represents perfect hip or knee health.

Patient electronic medical records were also retrospectively retrieved and analyzed to determine same-day discharge rate, overall readmission rates, and reason for admission as well as the number of other patient and surgical characteristics (EHR, Epic Systems, Verona WI). Other patient-specific variables included age, gender, race, American Society of Anesthesiologists score, weight, height, and body mass index. Surgery-related variables comprised the type of surgery, surgery laterality, anesthetic used, total procedure time, total anesthesia time, total care time, time to discharge, type of opioid used in the postanesthesia care unit, and the surgical approach (Table 1).

**Table 1:** Surgical approach utilized for total hip arthroplasty (THA), total knee arthroplasty (TKA), and unicondylar knee arthroplasty (UKA) procedures.

Surgical approach	No. of patients
THA	
Anterior	49
Lateral	17
TKA	
Medial parapatellar	22
Medial trivector	13
UKA	
Lateral parapatellar	37
Medial parapatellar	143

HOOS, JR, Hip Disability and Osteoarthritis Outcome Score of Joint Replacement; KOOS, JR, Knee Injury and Osteoarthritis Outcome Score for Joint Replacement.

### Demographic information

During the study time frame, there were 2075 THA, 2456 TKA, and 309 UKA or a total of 4840 TJA and UKA procedures completed. Of this population, 149 THA (0.7%), 73 TKA (2.9%), and 237 UKA (76.7%) or a total of 459 (9.4%) patients underwent same-day discharge. There were 8 patients who were designated as outpatient but ended up staying (5 THA, 3 TKA). Our survey response population consisted of 281 THA, TKA, and UKA procedures, including 66 (23.5%) same-day discharge THA, 35 (12.5%) TKA, and 180 (64.0%) UKA patients (Table 2). Of these, 122 (43.4%) patients identified as male and 159 (56.6%) patients identified as female. Twenty-nine (10.3%) patients were between 19 and 49 years of age, 130 (46.3%) patients were between 50 and 64 years of age, and 122 (43.4%) patients were 65 years or older. Patients were identified as outpatients if they were discharged on a same-day basis.

**Table 2:** Patient demographics and spinal anesthesia utilization.

Characteristic	Procedure types		
	THA	TKA	UKA
No.	66	35	180
Age of patient, y			
19-49	14	2	13
50-64	34	20	76
≥65	18	13	91
Sex of patient			
Male	23	17	82
Female	43	18	98
Spinal anesthesia			
Bupivacaine 0.5%	4	0	3
Bupivacaine 0.75% in dextrose 8.25%	2	1	7
Mepivacaine injection 1.5%	0	22	146
Ropivacaine injection 0.2%	0	1	1
Ropivacaine injection 0.5%	59	10	19

THA, total hip arthroplasty; TKA, total knee arthroplasty; UKA, unicondylar knee arthroplasty.

Patients qualified for same-day discharge on the basis of the following 6 criteria: (1) Patients must be medically safe and cleared for same-day discharge; (2) Patients must have someone able to stay with them overnight on the day of surgery; (3) Patients must attend an institutional preoperative joint education class; (4) Patients must achieve adequate pain control after their procedure; (5) Patients must be able to climb stairs as evaluated by a physical therapist after their procedure; and (6) Patients must consent to same-day discharge at the preoperative visit.

### Statistical analysis

Descriptive statistics were calculated using Excel (Microsoft, Redmond WA). Descriptive statistics include characteristic counts, mean values, standard deviations (SDs), and 95% confidence intervals (CIs). Multivariate linear regression modeling was next conducted to identify patient and surgical factors that could predict PROMs following THA, TKA, or UKA. The independent variables incorporated into our model encompassed both patient and surgical characteristics. A multiple linear regression analysis was conducted to identify significant predictors of PROMs, utilizing the ordinary least squares method. The analysis was performed using Python, version 3.8.12 (Python Software Foundation), with the statsmodels library, and categorical variables were appropriately encoded to ensure accurate interpretation. Model coefficients, standard errors, and *P* values were computed for each predictor variable. To address collinearity among the predictor variables, the variance inflation factor was calculated for each variable, with all values found to be well below 5. Statistical significance was set at 2-sided *P* < .05.

## Results

One hundred percent of THA, 93.8% of TKA, and 93.3% of UKA patients surveyed stated that they would elect to undergo their respective procedure again (patient satisfaction). Cumulatively, 94.6% of patients stated they would elect to repeat their TJA procedure. Furthermore, 94.3% of THA, 81.3% of TKA, and 95.6% of UKA patients stated they would like to be discharged on an outpatient basis again. Cumulatively, 92.7% of patients surveyed stated that they would like to be discharged on an outpatient basis.

THA patients reported a mean HOOS, JR score of 95.6 (SD 8.4, 95% CI 87.2-104.0), TKA patients reported a mean KOOS, JR score of 89.8 (SD 11.1, 95% CI 78.6-100.9), and UKA patients reported a mean KOOS, JR score of 86.3 (SD 16.4, 95% CI 69.9-102.7) (Table 3).

**Table 3:** Patients-reported outcomes after total hip arthroplasty (THA), total knee arthroplasty (TKA), or unicondylar knee arthroplasty (UKA) using the HOOS, JR and KOOS, JR surveys.

Question category	Patient-reported outcome				
	None	Mild	Moderate	Severe	Extreme
HOOS, JR Questions (THA)					
Hip pain when going up or down stairs	30	5	0	0	0
Hip pain when walking on uneven surface	31	4	0	0	0
Difficulty rising from sitting	31	4	0	0	0
Difficulty bending to floor/pick up an object	31	4	0	0	0
Difficulty lying in bed	30	5	0	0	0
Difficulty sitting	33	2	0	0	0
KOOS, JR Questions (TKA)					
Knee stiffness when waking up in the morning	8	6	2	0	0
Pain when twisting/pivoting on your knee	13	2	1	0	0
Pain when straightening knee fully	15	0	1	0	0
Pain when going up or down stairs	11	3	1	1	0
Pain when standing upright	14	1	1	0	0
Difficulty rising from sitting	15	1	0	0	0
Difficulty bending to floor/pick up an object	16	0	0	0	0
KOOS, JR Questions (UKA)					
Knee stiffness when waking up in the morning	22	13	6	3	0
Pain when twisting/pivoting on your knee	35	5	1	2	1
Pain when straightening knee fully	38	4	1	1	0
Pain when going up or down stairs	30	7	3	3	1
Pain when standing upright	38	3	1	1	0
Difficulty rising from sitting	33	6	2	2	0
Difficulty bending to floor/pick up an object	34	6	0	3	0

HOOS, JR, Hip Disability and Osteoarthritis Outcome Score of Joint Replacement; KOOS, JR, Knee Injury and Osteoarthritis Outcome Score for Joint Replacement.

The mean time to discharge was 5.4 hours (SD 1.4, 95% CI, 4.0-6.8) for THA, 4.9 hours (SD 1.6, 95% CI, 3.3-6.5) for TKA, and 4.7 hours (SD 1.6, 95% CI, 3.1-6.3) for UKA. There were a total of 2 readmissions in our patient cohort, with no repeated readmissions. The readmission rates for THA, TKA, and UKA patients were 1.5%, 0.0%, and 0.5%, respectively. One THA patient was readmitted due to joint infection and underwent surgical management, and 1 UKA patient was readmitted due to constipation and urinary retention.

Multivariate linear regression of 11 patient and surgical factors did not yield any significant predictors of HOOS, JR or KOOS, JR interval scores (Table 4). The variables most positively correlated with the HOOS, JR or KOOS, JR interval scores were the type of anesthetic used and time to discharge. However, none of these correlations were statistically significant.

**Table 4:** Multivariate linear regression analysis of patient and surgical characteristics predicting HOOS, JR or KOOS, JR interval scores.

Variable	Coefficient	Standard error	*P* value
Patient	−0.03	0.07	.63
Age	−0.16	0.17	.36
Gender	1.55	3.39	.65
Surgery type (THA, TKA, UKA)	0.47	2.27	.84
Race	−0.34	2.17	.88
Laterality (L,R)	4.08	3.35	.23
BMI	0.08	0.39	.84
Spinal anesthetic	3.37	1.99	.10
Total care time	−0.01	0.02	.69
Time to discharge from PACU phase I	0.03	0.03	.19
Opioid used in PACU	1.05	1.01	.31

BMI, body mass index; HOOS, JR, Hip Disability and Osteoarthritis Outcome Score of Joint Replacement; KOOS, JR, Knee Injury and Osteoarthritis Outcome Score for Joint Replacement; PACU, postanesthesia care unit; THA, total hip arthroplasty; TKA, total knee arthroplasty; UKA, unicondylar knee arthroplasty.

## Discussion

Outpatient surgery is a viable choice for clinicians and patients alike and can significantly decrease healthcare expenditures, as outpatient TKA is estimated to save as much as US $8527 compared to a postoperative inpatient stay of 3-4 days [13]. This is reflected in the growing trend toward performing joint arthroplasty on a purely outpatient basis, with a 15.8% mean annual outpatient procedure volume increase for THA and 11.1% increase for TKA between 2010 and 2017 [14]. Previous studies have evaluated patient satisfaction in the ASC setting, but this is the first study to determine patient satisfaction in outpatient joint arthroplasty in AMCs. Our study found a cumulative self-reported satisfaction rate of 94.6%, with stratified rates of 100% for THA, 93.8% for TKA, and 93.3% for UKA patients, which was comparable to the ASC setting. Moreover, a cumulative 92.7% of patients stated their desire to be discharged as an outpatient again, with stratified rates found to be 94.3% for THA, 81.3% for TKA, and 95.6% for UKA patients.

For patients, the choice of receiving their outpatient arthroplasty in either an ASC or AMC may be influenced by the growing popularity of ASCs, surgery costs, highly specialized and smaller care teams in ASCs and convenience factors [15]. In a single ASC study, Berend et al [5] reported a high patient satisfaction rate of 98%-100% and 2% readmission rate among a cohort of 1230 outpatient TJA patients across a 2-year period, to which they attribute to highly specialized care and lower regulatory demands that are perhaps more amenable in an ASC. In addition to increased patient satisfaction, ambulatory surgery centers are actively reducing healthcare spending on outpatient procedures [16]. For example, it has been shown that the use of an ambulatory surgery center for orthopedic day surgery results in as much as 17%-43% direct cost savings when compared to an AMC. This difference is due, in large part, to the increased difficulties and costs of setting up outpatient surgeries in AMCs when compared to ASCs. AMCs have higher caseloads, operating room booking limitations, rotating trainees, and provider variability. Furthermore, staff at AMCs often cover different specialties, leading to difficulties with operating room coverage. Given that the transition to outpatient surgery can significantly reduce medical expenditure with no increase in complications, AMCs should focus on addressing these barriers [17]. However, an important advantage of conducting TJA procedures at AMCs is the ability to easily convert outpatients into inpatient admissions if needed for observation.

Additionally, we sought to determine surgical procedure outcomes following outpatient THA, TKA, or UKA in AMCs. The mean score for THA patients on the HOOS, JR was 95.6, which is very consistent with similar studies; one analysis using 1281 THA patients found the mean HOOS, JR score of 95.5 at 6 months and 94.1 at 12 months [18]. However, for total and partial knee arthroplasty, patient satisfaction was found to be higher than prior reports. In our cohort, TKA and UKA patients scored an 89.8 and 86.3 on the KOOS, JR, respectively, compared to prior studies which reported KOOS, JR scores in the 56.5-60.3 range in adult reconstruction patients [19]. Given that hip and knee function are critical for activities of daily living, it is important to continue to trend these outcome measures. Moreover, the increased age-standardized rates for disability-adjusted life years lost to hip and knee dysfunction have led to an urgent need for institutional response by large AMCs [20].

We also evaluated the average time to discharge for the THA, TKA, and UKA patients. The mean time to discharge for the 3 cohorts was 5.4 hours (SD 1.4, 95% CI, 4.0-6.8), 4.9 hours (SD 1.6, 95% CI, 3.3-6.5), and 4.7 hours (SD 1.6, 95% CI, 3.1-6.3), respectively. This corroborated the findings of other studies, which found the average time to discharge after an outpatient TJA to be between 4 and 8 hours [21]. However, 1 retrospective review of 1743 primary TJA patients at a community hospital found an average time to discharge of 12.3 hours and 11.8 hours, for TKA and THA, respectively [22]. Literature has shown that decreasing time to discharge after joint arthroplasties is a useful strategy for minimizing healthcare costs, which is particularly relevant given the projected upward trend in joint arthroplasty utilization in the coming years [23]. Kurtz et al [24] have projected the demand for primary TKA and THA in patients younger than 65 years to increase by more than 50% between 2010 and 2030, highlighting the need for AMCs to transition to outpatient care.

Recent studies comparing complication trends in outpatient vs nonoutpatient THA, TKA, and UKA found no differences in overall adverse events or readmission between same-day and inpatient groups [25]. In a study of 1,789,601 patients between 2010 and 2017, Debbi et al [14] have shown that there was no significant difference for outpatient TKA or THA vs nonoutpatient TKA or THA. Similarly, Basques et al [26] demonstrated no significant differences in overall postoperative complications or readmissions in TKA or THA patients between inpatient and outpatient groups. Additionally, Reddy et al observed no difference in risk for severe adverse events such as deep infection, venous thromboembolism, and mortality when comparing same-day THA and inpatient stays for THA. Thus, the orthopaedic literature supports the notion that outpatient TJA procedures are equally safe as TJA procedures done with inpatient admission.

A systematic review of outpatient TJA at ASCs by Rodríguez-Merchán et al cited TKA and UKA readmission rates of 3.6% at ASCs and additionally stated that readmission rates in patients discharged in an outpatient basis were similar to matched patients with at least 1 overnight stay [13]. These findings are in line with our data, which demonstrate a cumulative readmission rate of 0.7% (1.5% THA, 0.0% TKA, 0.5% UKA). Importantly, our data suggest that outpatient TJA at AMCs may yield similar or lower rates of readmissions to TJA procedures carried out at ASCs. Furthermore, this readmission rate is the same as or lower than readmissions rates for traditional TKA and THAs determined by analyses of Medicare fee-for-service data reports [27,28]. For example, Middleton et al [29] reported that an analysis of outcomes in Medicare beneficiaries showed readmissions rates of 6.3% for knee arthroplasty, 7.0% for elective hip arthroplasty, and 19.2% for nonelective hip arthroplasty. An investigation by Cram et al [30] examining an observation cohort of 3,271,851 patients undergoing TKA over a 20-year period from 1991 to 2010 demonstrated an all-cause 30 day readmission rate of 5.0%, which is similar to our reported TKA readmission rate. Similar work in spine surgery by Safaee et al [31] investigating the difference in complication rates, readmission rates, and emergency department visits in patients undergoing discectomy procedures demonstrated no differences between care received at AMCs and ASCs. Our institutional data are thus consistent with the literature that outpatient orthopedic procedures at AMCs are as safe as inpatient AMC procedures as well as outpatient ASC procedures.

We next conducted a multiple linear regression analysis to determine the influence of several patient and surgical characteristics on PROMs. Our model incorporated a broad array of predictors, including patient demographics, surgical details, and types of postoperative medications used. However, the results of our analysis did not yield any statistically significant predictors of PROMs. This is most likely due to the fact that recovery following THA, TKA, or UKA is multifactorial and could be influenced by a combination of variables not included in our model, such as rehabilitation following surgery or individual comorbidities that can impact recovery. Future studies should aim to incorporate a wider range of predictors and consider the use of more advanced modeling techniques to enhance the predictability of PROMs following outpatient TJA.

This study has several limitations. Data used in this study were gathered from 2 AMCs in the Northeast United States, which limit the sample size and the generalizability of the results. Additionally, only 9.4% of all TJA patients during the study period underwent same-day discharge, indicating a possible selection bias in patient selection for outpatient TJA. This bias was more pronounced in the THA and TKA patient groups than in UKA patients. This may indicate that only a select group of patients can undergo successful THA or TKA, whereas a broader population may successfully undergo UKA. Furthermore, because patients were allowed to freely decide which survey questions they answered, the survey response numbers varied. Another limitation of our study is that the HOOS, JR and KOOS, JR surveys are not globally utilized instruments, but they do provide patient self-assessments of surgical outcomes that can only be determined by survey questionnaires. Finally, our data are biased toward a higher number of UKAs as compared to TKAs or THAs, which may impact our linear regression model and statistical conclusions.

## Conclusions

While complex factors influence both patient satisfaction and outcomes following TJA or UKA, the present study found that patients who underwent outpatient joint arthroplasty procedures at 2 AMCs reported a high desire to repeat their outpatient procedures, had high PROMs, had short times to discharge and experienced minimal readmissions. In order to better assess these outcomes, future studies should be focused on gathering a larger patient sample from other AMCs to assess these PRO parameters. Additional studies could also compare HOOS, JR or KOOS, JR scores of outpatient vs inpatient TJA or UKA patients, along with PROMs in outpatient TJA or UKA patients in ASCs vs AMCs.

## Appendix

1. HSS HOOS, JR Survey: https://www.hss.edu/files/hss-hoos-jr.pdf

2. HSS HOOS, JR Scoring Instructions: https://www.hss.edu/files/HOOS-JR-Scoring-Instructions-2017.pdf

3. HSS KOOS, JR Survey: https://www.hss.edu/files/hss-koos-jr.pdf

4. HSS KOOS, JR Scoring Instructions: https://www.hss.edu/files/KOOS-JR-Scoring-Instructions-2017.pdf

5. Patient Satisfaction Survey:

Q1. What kind of joint replacement surgery did you undergo?

Q2. When was your joint replacement done?

Q3. Were you satisfied with this procedure and would you undergo this procedure again?

Q4. Did you go home the same day as your surgery?

If yes:
Q4.1. Would you want to be discharged home the same day again?

Q5. Did you return to the hospital after the surgery for any reason that was related to the surgery?

If yes:
Q5.1. When did you return to the hospital?
Q5.2. Did you go to the emergency room only or were you admitted to the hospital?
Q5.3. What were you admitted for?
Q5.4. Did you undergo surgery again?

## Appendix A Supplementary data

Conflict of Interest Statement for Chen

Conflict of Interest Statement for Duggan

Conflict of Interest Statement for Ghoshal

Conflict of Interest Statement for Howell

Conflict of Interest Statement for Salazar

Conflict of Interest Statement for Shah
