# Supplementary material for: Assessment of Patient Satisfaction and Outcomes After Outpatient Joint Arthroplasty in Academic Medical Centers
Source: Arthroplast Today. 2023 Nov 10;24:101246. doi: 10.1016/j.artd.2023.101246 (PMC10776316; doi:10.1016/j.artd.2023.101246)
Supplement: Conflict of Interest Statement for Chen [file mmc1.pdf]

Combined CONFLICT OF INTEREST STATEMENT

The Journal of Arthroplasty

(Adopted from the American Academy of Orthopaedic Surgeons disclosure statement)

The following form **must be filled out completely and submitted by each author (example, 6 authors, 6 forms)**. **If no discloser is required, please write/type "none" at the end of each sentence.**

Manuscript Title Assessment of Patient Satisfaction and Outcomes Following Outpatient Joint Arthroplasty in Academic Medical Centers

1. Royalties from a company or supplier (The following conflicts were disclosed)
Stryker

2. Speakers bureau/paid presentations for a company or supplier (The following conflicts were disclosed)
None

3A. Paid employee for a company or supplier (The following conflicts were disclosed)
None

3B. Paid consultant for a company or supplier (The following conflicts were disclosed)
Adaptive Phage Therapeutics, Avanos, BICMD, Convatec, Ethicon, GLG, Guidepoint, Heraeus, IrriMax, Pfizer, Stryker

3C. Unpaid consultants for a company or supplier (The following conflicts were disclosed)
None

4. Stock or stock options in a company or supplier (The following conflicts were disclosed)
Hyalex, Irrimax, Joint Purification Systems, Sonoran, IlluminOss

5. Research support from a company or supplier as a Principal Investigator (The following conflicts were disclosed)
None

6. Other financial or material support from a company or supplier (The following conflicts were disclosed)
None

7. Royalties, financial or material support from publishers (The following conflicts were disclosed)
SLACK Incorporated, UpToDate

8. Medical/Orthopaedic publications editorial/governing board (The following conflicts were disclosed)
Journal of Arthroplasty; Clinical Orthopaedics and Related Research; Journal of Bone and Joint Infection; Journal of Bone and Joint Surgery; Arthroplasty Today

9. Board member/committee appointments for a society (The following conflicts were disclosed)
AAOS, AJRR, AAHKS

Each author must sign AND print or type his/her name, date and submit a separate form

In addition, one BLINDED Conflict of Interest form (no author names used) should be submitted per manuscript with all author disclosures.

Antonia F. Chen, MD/MBA	Antonia Chen	11/29/2022
Author Name (Print or Type)	Author Signature	Date
